# MMP19 Is Essential for T Cell Development and T Cell-Mediated Cutaneous Immune Responses

**DOI:** 10.1371/journal.pone.0002343

**Published:** 2008-06-04

**Authors:** Inken M. Beck, René Rückert, Katja Brandt, Markus S. Mueller, Thorsten Sadowski, Rena Brauer, Peter Schirmacher, Rolf Mentlein, Radislav Sedlacek

**Affiliations:** 1 Institute of Biotechnology, Prague, Czech Republic; 2 Institute of Molecular Genetics, Prague, Czech Republic; 3 Research Center Borstel, Department of Immunology and Cell Biology, Borstel, Germany; 4 Swiss Tropical Institute, Basel, Switzerland; 5 Sanofi-Aventis Deutschland GmbH, Frankfurt, Germany; 6 Department of Biochemistry, University of Kiel, Kiel, Germany; 7 Institute of Pathology, University Hospital Heidelberg, Heidelberg, Germany; 8 Department of Anatomy, University of Kiel, Kiel, Germany; Centre de Recherche Public-Santé, Luxembourg

## Abstract

Matrix metalloproteinase-19 (MMP19) affects cell proliferation, adhesion, and migration *in vitro* but its physiological role *in vivo* is poorly understood. To determine the function of MMP19, we generated mice deficient for MMP19 by disrupting the catalytic domain of *mmp19* gene. Although MMP19-deficient mice do not show overt developmental and morphological abnormalities they display a distinct physiological phenotype. In a model of contact hypersensitivity (CHS) MMP19-deficient mice showed impaired T cell-mediated immune reaction that was characterized by limited influx of inflammatory cells, low proliferation of keratinocytes, and reduced number of activated CD8^+^ T cells in draining lymph nodes. In the inflamed tissue, the low number of CD8^+^ T cells in MMP19-deficient mice correlated with low amounts of proinflammatory cytokines, especially lymphotactin and interferon-inducible T cell α chemoattractant (I-TAC). Further analyses showed that T cell populations in the blood of immature, unsensitized mice were diminished and that this alteration originated from an altered maturation of thymocytes. In the thymus, thymocytes exhibited low proliferation rates and the number of CD4^+^CD8^+^ double-positive cells was remarkably augmented. Based on the phenotype of MMP19-deficient mice we propose that MMP19 is an important factor in cutaneous immune responses and influences the development of T cells.

## Introduction

Matrix metalloproteinases (MMPs) are zinc-dependent endopeptidases that are responsible for processing extracellular matrix (ECM) proteins and a number of cell surface receptors, their ligands, and adhesion molecules. These proteolytical activities have direct impact on cell behavior, proliferation, and survival (reviewed in [1]–[3]).

MMP19 was originally isolated from the inflamed synovium of a rheumatoid arthritis patient [4], from mammary gland, and liver [5], [6]. Human and murine orthologues of MMP19 (human: U37791, murine: AF153199) retain the common domain organization of soluble members of the MMP family, however, they also contain several distinctive features including the insertion of a unique cysteine in the catalytic domain, an altered latency motif, a unique oligoglutamate insertion in the hinge region, and a C-terminal tail [4]–[9]. At the mRNA level, MMP19 is expressed in many tissues [5], [6]. However, the expression at the protein level appears to be more restricted. Vascular smooth muscle cells, myoepithelial cells, and basal keratinocytes express MMP19 constitutively whereas endothelial cells, epithelial cells of the mammary glands, and monocytes show differential regulation of this enzyme [10]–[15]. In addition to its characteristic expression pattern in the vicinity of basement membranes, MMP19 is able to cleave extracellular matrix (ECM) components such as type IV collagen, γ2 chain of laminin 5, nidogen-1, the large tenascin-C isoform, fibronectin, cartilage oligomeric matrix protein (COMP), and aggrecan [9], [16]–[18]. Moreover, MMP19 effectively cleaves insulin-like growth factor binding protein-3 (IGFBP-3) as we showed previously [14]. MMP19 was also reported to be expressed in macrophages [15], [19] and upregulated under inflammatory conditions such as arthritis and multiple sclerosis [20]–[22]. Recently, it became obvious that beside their role in matrix processing MMPs are important in the development and outcome of inflammatory responses by activating and releasing cytokines, establishing a chemokine gradient, controlling cell migration and survival, and contributing to epidermal barrier function [23].

To reveal specific functions of an MMP in a cutaneous immune response as well as in the epidermis, immune reactions of delayed-type hypersensitivity (DTH) are especially useful due to their well characterized course. Contact hypersensitivity (CHS) represents a subtype of DTH reactions in the skin that is used as model for allergic contact dermatitis mediated by T lymphocytes [24], [25]. Upon cutaneous contact with haptens, small molecules, which act as antigen, T lymphocytes are activated by Langerhans cells, i.e. epidermal dendritic cells (DCs). Antigen-bearing DCs are targeted to regional lymph nodes (LN) and present the antigen to activate and clonally expand specific T cell precursors. During the efferent phase, after secondary exposure to the hapten both CD4^+^ and CD8^+^ T cells are recruited into the skin. CD8^+^ T cells appear to be key effectors of CHS, since depletion of CD8^+^ T cells reduce CHS while MHC class II-deficiency leads to enhanced response [26]–[31]. CD4^+^ T cells have a prominent role in the resolution and downregulation of CHS [24], [32], [33].

Hapten application induces secretion of cytokines by various cell types in the skin. Involvement of interleukin-1 (IL-1), tumor necrosis factor α (TNF-α), and monocyte chemoattractant protein-1 (MCP-1) during early stage of sensitization has been reported [34]–[38]. For T cell activation and generation of memory T cells promoting cytokines like IL-16, MCP-1, MIF, and IL-2 are of crucial importance [39]–[43]. During the elicitation phase of CHS at the site of hapten challenge, cytokines such as TNF-α, interferon-γ (IFN-γ), growth-related oncogene-α (Gro-α), MCP-1, and RANTES (regulated and normal T cell expressed and secreted) recruit neutrophils and eosinophils while lymphotactin and I-TAC are responsible for the activation and recruitment of T cells [38], [44]–[53].

To exercise a control function in the periphery, effector T cells that are responsible for immune reactions such as CHS have to undergo a proper and accurate development and distribution. In the thymus T lymphocytes develop from early thymocyte progenitors to differentiated, functional T cells that emigrate to the periphery. During their maturation CD4 and CD8 single-positive (SP) mature T lymphocytes develop through differentiation stages involving CD4^−^CD8^−^ double-negative (DN) and CD4^+^CD8^+^ double-positive (DP) thymocytes. DP thymocytes that fail the selection process or are negatively selected die at this stage. Positive selection leads to transition into SP thymocytes and emigration of mature T cells into periphery. Throughout the maturation process thymocytes have to migrate through cortical and medullary regions along ECM components like laminins, fibronectin, and collagen IV [54], which in part is mediated by the action of MMPs [55].

To analyze the function of MMP19 *in vivo* mice deficient in *mmp19* gene expression were generated via targeted mutagenesis. Since MMP19 is abundantly expressed in the epidermis and is upregulated under inflammatory conditions we focused our investigations onto immune reactions in the skin. To study the effect of MMP19 in T cell-mediated immune responses we used fluorescein isothiocyanate (FITC) as a hapten in the model of CHS and analyzed activation of DCs and CD8^+^ T cells. We show here that MMP19-deficient mice exhibited a limited inflammatory reaction in response to the hapten marked by lowered cytokine expression, decreased influx of inflammatory cells, low proliferation of keratinocytes, and diminished activation and distribution of CD8^+^ T cells. Since differences or depletion of T cell subsets could alter the CHS response [26], 27, 29, 56 we further analyzed T cell populations in the blood and thymus under normal unsensitized conditions. Relative numbers of single-positive CD4^+^ and CD8^+^ T cells were reduced in the blood and thymus whereas the number of CD4^+^CD8^+^ double-positive T cells in the thymus was remarkably augmented. Thus, MMP19 is an important factor, which is necessary for proper immune response in the skin and for development and distribution of T cell populations.

## Results

### Generation of MMP19-deficient mice

**Figure 1:**
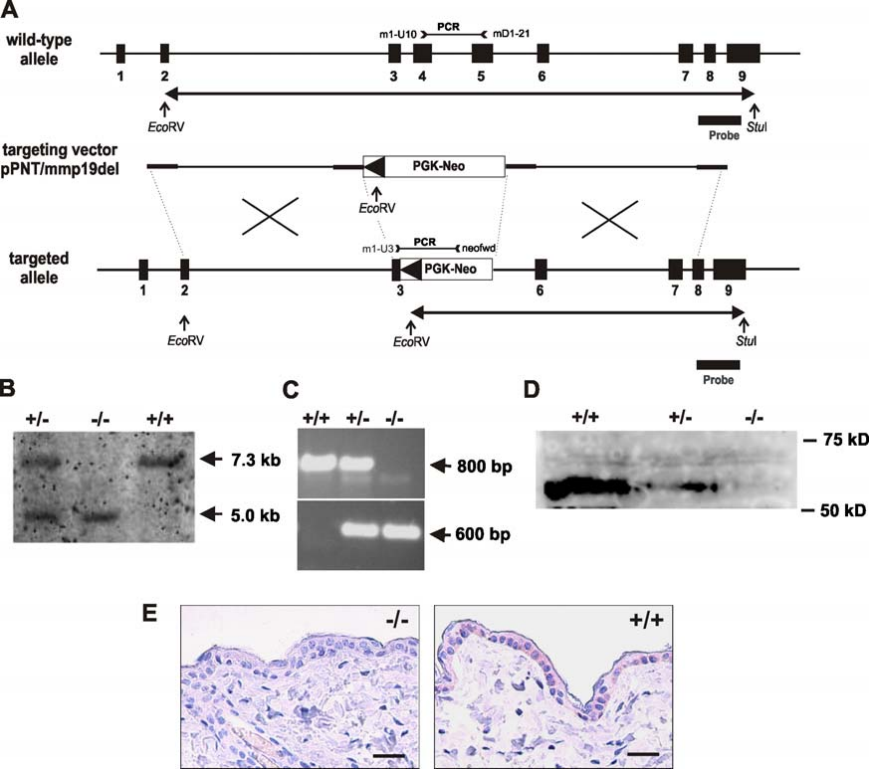
Generation of MMP19^−/−^ mice. (A) Schematic representation of the murine *mmp19* gene and its exon/intron-organization as previously described by Mueller et al. [7]; pPNT targeting vector construct, and the resulting deleted active site locus of the mouse *mmp19* gene are depicted. The targeting construct based on the pPNT vector [70], was generated by replacement of 1088 bp region of *mmp19* gene spanning the end of exon 3 and the whole exon 4 encoding the catalytic domain by the neomycin resistance cassette. Homologous recombination led to introduction of the PGK-Neo cassette and allowed selection of homologous recombinants. The 3′-probe used for detection of replacement events is indicated by thick bars. Also shown are restriction sites used for southern hybridization screening as well as primer binding sites used for diagnostic PCR. Screening for replacement mutants employed restriction digestion of genomic DNA with *Stu*I and *Eco*RV and southern hybridization with the described 3′-probe. (B) For screening of MMP19-deficient mice Southern blot analyses were performed: mouse DNA digested with *Stu*I and *Eco*RV was probed with the diagnostic 3′-probe. Probing led to identification of either a 7.3 kb band (wild-type allele, +/+) or a 5 kb band for the targeted allele (−/−). In heterozygous mice (+/−) both alleles are present. (C) Genotyping of targeted alleles using PCR. Wild-type alleles are detected by an 800 bp band, while PCR for the MMP19-deficient allele results in a 600 bp product. (D) Primary keratinocytes isolated from wild-type, heterozygous, and homozygous MMP19-deficient mice were analyzed for MMP19 expression by western blotting using anti-MMP19 antibodies purified against a peptide derived from the hinge region of murine MMP19, that is deteced in size of 59 kD. (E) Immunohistochemical analysis of murine skin with anti-MMP19 antibodies described above. Scale bars: 50 µm.

In order to assess the physiological function of MMP19, we constructed a targeting vector that replaced the catalytic domain with a neoR cassette orientated in the opposite direction to the endogenous *mmp19* gene (Fig. 1A). To determine mice carrying the targeting construct Southern blot analysis and PCR of DNA from newborn mice were carried out (Fig. 1B and C). The absence of MMP19 protein in MMP19-deficient (MMP19^−/−^) mice was demonstrated by immunoblotting of cell lysates prepared from MMP19^−/−^, heterozygous (MMP19^+/−^), and wild-type primary keratinocytes (Fig. 1D). The absence of MMP19 at the protein level in MMP19^−/−^ mice was also shown by immunohistochemical analysis of murine skin with antibodies directed against the hinge region of mouse MMP19 (Fig. 1E).

Mice homozygous for the targeted gene were observed for more than one year. They developed without obvious alteration in gross morphology, weight, size, and fecundity compared to wild-type mice. Breeding mice heterozygous for the targeted gene produced the expected Mendelian ratio of knockout animals of 25%. Thus, MMP19 does not seem to be essential for blastocyst implantation or embryonic development. No apparent changes in the histology of brain, bones, heart, kidney, liver, lung, reproductive organs, pancreas, skin, and spleen of MMP19-deficient mice were seen at 1, 3, 6, and 12 months (data not shown).

### MMP19^−/−^ mice exhibit impaired CHS

**Figure 2:**
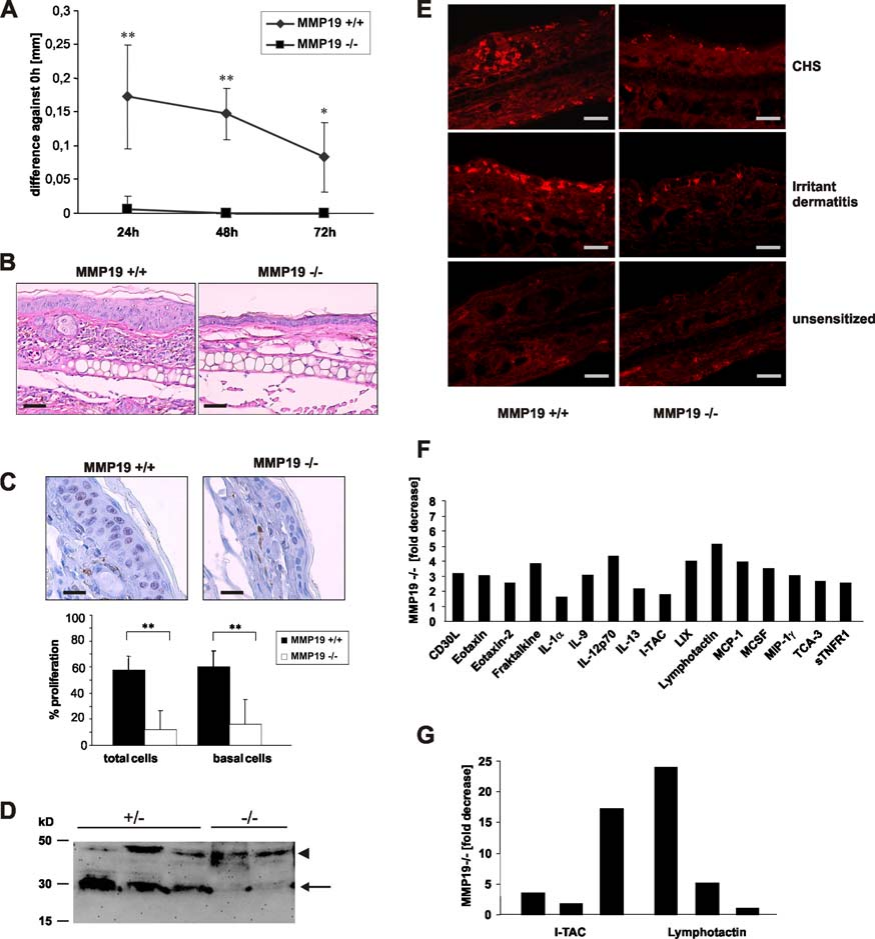
MMP19^−/−^ mice show impaired ear swelling and inflammatory reaction in CHS. (A) Five days after abdominal sensitization with the hapten (FITC), mouse ears were challenged with FITC and ear thickness was measured after 24, 48, and 72 h. Mean values are given in mm as difference to time point 0 h. (B) Hematoxylin-eosin staining of ear sections 24 h after challenge shows reduced influx of neutrophils and eosinophils in MMP19^−/−^ mice. (C) Staining with anti-Ki-67 antibody revealed that proliferation of keratinocytes in MMP19-deficient mice was strongly reduced in basal and suprabasal layers. Ki-67-positive cells were counted and calculated as percentage of basal and total keratinocytes. Six images (magnification 400x) per mice were analyzed. (D) Decreased processing of IGFBP-3 in MMP19-deficient mice. Primary keratinocytes from MMP19^+/−^ and MMP19^−/−^ mice were grown for 72 h and conditioned media were analyzed for IGFBP-3 proteolysis by western blotting. The arrowhead indicates the position of intact IGFBP-3, whereas the arrow points to 30 kD IGFBP-3 proteolytic fragment. (E) Anti-CD8 staining (red) of ear sections. MMP19^−/−^ mice show low numbers of CD8^+^ T cells in CHS (upper panel) as well as in a T cell-independent model of inflammation, i.e. irritant dermatitis, induced by croton oil (middle panel). Ears of unsensitized mice (lower panel) exhibit low numbers of CD8^+^ cells; no difference was observed between MMP19^+/+^ and MMP19^−/−^ mice. CHS and irritant dermatitis were carried out in four independent experiments each with 4 wild-type and 4 MMP19^−/−^ mice. (F) 24 h after FITC challenge ear lysates were analyzed for cytokine expression that was generally reduced in MMP19-deficient mice compared to wild-type animals. (G) Reduced levels of lymphotactin and I-TAC from three independent experiments are shown. Bars in F and G represent values of MMP19^−/−^ mice given as fold decrease to wild-type mice. Significant values with *p<0.05 and **p<0.01; student's t-test. Scale bars: B, 50 µm; C, 20 µm; D, 50 µm.

To investigate the role of MMP19 in immune responses MMP19^−/−^ mice and wild-type control mice were studied in a model of CHS. In order to follow up the activation and migration of DCs in the first stage of CHS, FITC was used as a fluorogenic hapten that facilitates the detection of activated DCs, i.e. FITC-positive DCs, in lymph nodes by flow cytometry. No significant differences in the number or activation (CD80, CD86, MHC class II expression) of DCs could be detected between MMP19^−/−^ and MMP19^+/+^ mice (not shown). After cutaneous sensitization and challenge of the mice by painting FITC on one ear, wild-type mice developed a local inflammatory response that was measured as an increase of ear thickness with a peak around 24 h after challenge (Fig. 2A). This swelling declined slowly during the period of three days. In contrast, ear swelling was abrogated in MMP19-deficient mice (Fig. 2A). Histology of ears 24 h post FITC challenge revealed strong infiltration of inflammatory cells in MMP19^+/+^ mice that was not observed in MMP19^−/−^ mice (Fig. 2B). The inflammatory reaction in wild-type mice was accompanied by a strongly increased proliferation of keratinocytes resulting in a thickened epidermis. The activation and thickening of the epidermis were absent in MMP19^−/−^ mice (Fig. 2B and C). Evaluation of keratinocytes positive for the proliferation marker Ki-67 showed that 60% of total and basal keratinocytes were proliferating in MMP19^+/+^ mice while in MMP19-deficient mice only 20% of keratinocytes proliferated (Fig. 2C). Since MMP19 is known to affect keratinocyte proliferation by processing IGFBP-3 [14], we isolated primary mouse keratinocytes from MMP19^−/−^ and wild-type mice and compared their capacity to process IGFBP-3 using western blot analysis. While keratinocytes from MMP19^+/−^ mice exhibited usual degradation of IGFBP-3, keratinocytes deficient for MMP19 showed predominantly the unprocessed form of IGFBP-3 (Fig. 2D).

Since FITC-stimulated CHS response is mainly mediated by CD8^+^ T cells [57], [58], we determined the presence of CD8^+^ cells in ears of wild-type and MMP19^−/−^ mice 24 h after FITC challenge as well as in unsensitized mice. In contrast to wild-type mice with abundantly dispersed CD8^+^ T cells in the challenged ear MMP19^−/−^ mice showed low numbers of T cells that were comparable to the unsensitized situation (Fig. 2E). To test whether the distribution of CD8^+^ T cells in MMP19^−/−^ mice differs from that of wild-type mice also under non-T cell-dependent inflammation, mouse ears were treated by a single painting of croton oil as unspecific, inflammatory stimulus. After 24 h, abundance of CD8^+^ T cells in MMP19-deficient mice was very low compared to wild-type mice (Fig. 2E). These findings indicate that MMP19^−/−^ mice have a defect in T cell-dependent inflammation, keratinocyte proliferation, and CD8^+^ T cell circulation.

### MMP19^−/−^ mice exhibit reduced production of inflammatory cytokines at the site of inflammation

Within inflamed tissues chemokines serve as main attractants for monocytes and T lymphocytes. Using a cytokine antibody array we compared ears of wild-type and MMP19^−/−^ mice for cytokine expression 24 h after FITC challenge. Generally, MMP19^−/−^ mice showed a reduction of all detectable cytokines when compared to MMP19^+/+^ mice. Fig. 2F shows a representative evaluation of these analyses with highest reduction in lymphotactin (5.1-fold) and IL-12 expression (4.3-fold). Other cytokines such as CD30L, eotaxin, fractalkine, LIX (LPS-induced chemokine), MCP-1, and MCSF (macrophage colony stimulating factor) were moderately reduced. IFN-γ, IL-4, and IL-5 were not detectable in these assays. Interestingly, lymphotactin and I-TAC, both responsible for activation and recruitment of T cells, showed continuously lower expression in MMP19^−/−^ mice (Fig. 2G). These results correspond with the previous analyses showing low inflammatory response in MMP19^−/−^ mice and point to a biased distribution of T cells at the inflammatory site.

### MMP19-deficiency results in reduced activation of T cells

**Figure 3:**
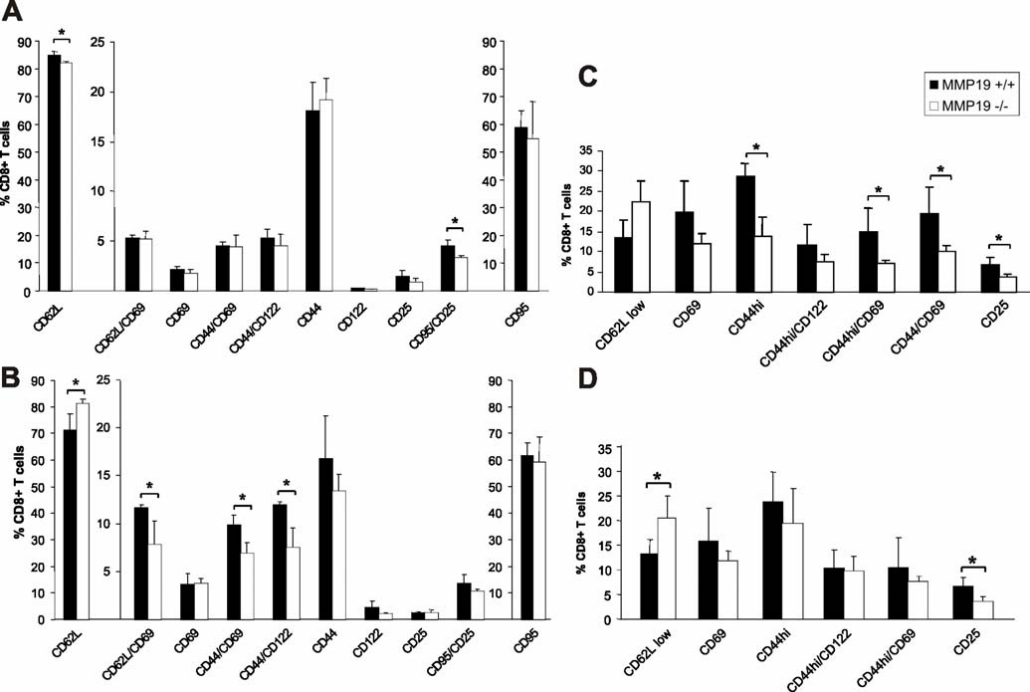
MMP19^−/−^ mice exhibit reduced T cell activation in CHS. Using flow cytometry cells of inguinal (A and B) or draining lymph nodes (C and D) from wild-type (+/+) and MMP19-deficient (−/−) mice were analyzed for the indicated activation markers, all gated on CD8^+^ T cells. (A) Unsensitized MMP19^−/−^ mice show slight decrease of T cells positive for CD62L and CD95/CD25. (B) MMP19^−/−^ mice analyzed 24 h after abdominal FITC painting (sensitization) exhibit higher numbers of naive T cells (CD62L^+^) and decreased numbers of activated T cells compared to MMP19^+/+^ mice. (C) In CHS reaction 48 h after FITC challenge on ears, draining lymph nodes of MMP19^−/−^ mice show significantly reduced numbers of cells positive for activation and memory markers. (D) Reduced numbers of CD25^+^ and high numbers of CD62L^+^ cells are still present in MMP19^−/−^ mice after 72 h while other activation markers were comparable to those of MMP19^+/+^ mice. In A and B two scales are used to match relevant data from an identical experiment. Significant values (p<0.05) are marked by asterisk. Each analysis was carried out four times with MMP19^+/+^ (n = 4) and MMP19^−/−^ mice (n = 4) per experiment.

Development of CHS depends on the stimulation and activation of antigen-specific T cells. We analyzed CD8^+^ T cells in unsensitized and FITC-sensitized mice (i.e. in the afferent phase) as well as in FITC-challenged mice with an active CHS response in the efferent phase, 48 and 72 h after treatment with the hapten. Unsensitized MMP19^−/−^ mice exhibited slightly reduced amounts of CD62L^+^ and CD25^+^/CD95^+^ cells in inguinal LN (Fig. 3A). Cells positive for CD69, CD44, and CD122 showed no differences under unsensitized conditions. 24 h after FITC-sensitization, i.e. abdominal hapten application, wild-type mice showed activation of T cells with increased amount of CD69^+^ and reduction of CD62L^+^ cells. In contrast, CD8^+^ T cells in inguinal lymph nodes of MMP19-deficient mice exhibited generally reduced activation (Fig. 3B). Numbers of activated CD8^+^ T cells positive for CD62L/CD69, CD44/CD69, and CD44/CD122 were significantly reduced compared to wild-type mice whereas the number of naive CD62L^+^ cells remained similar to the unsensitized situation indicating lack of activation (Fig. 3A). Five days after FITC sensitization, i.e. after memory T cells were generated, mice ears were challenged with FITC to induce CHS reaction. 24 h after challenge in correlation to the maximal inflammatory ear swelling response, the population of activated CD8^+^ T cells in the draining lymph nodes (DLN) of MMP19^−/−^ mice was strikingly diminished. Significant differences between MMP19^−/−^ and wild-type mice were observed for CD8^+^ T cells expressing CD44^high^, CD44/CD69, and CD25 (Fig. 3C). Although 72 h after FITC challenge MMP19^−/−^ mice did not show any inflammatory signs in the ears, CD8^+^ T cells in DLN from MMP19^−/−^ mice showed still some differences: the number of CD62L^+^ cells was increased and CD25^+^ cells diminished (Fig. 3D). We also analyzed DCs in DLN and observed no significant differences in CD80, CD86, CD40, and MHC class II positive cells between MMP19^−/−^ and wild-type mice (not shown). These results point out a significant role of MMP19 in T cell activation as well as in T cell distribution in peripheral tissues.

### Immature MMP19^−/−^ mice show low numbers of CD4^+^ and CD8^+^ T cells in peripheral blood

**Figure 4:**
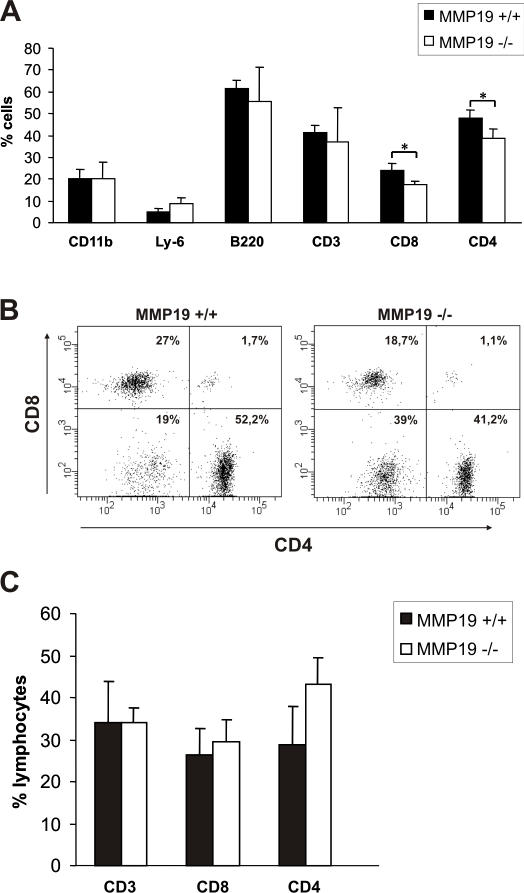
T cell population in blood of immature MMP19^−/−^ mice is distorted. (A) Blood samples of 3-weeks old MMP19^+/+^ and MMP19^−/−^ mice were analyzed for monocytes, granulocytes (CD11b and Ly-6), B cells (B220), and T cell populations by flow cytometry. MMP19^−/−^ mice exhibit significantly reduced numbers of CD4^+^ and CD8^+^ T cells. (B) A typical dot plot analysis of CD4^+^ and CD8^+^ populations gated on CD3^+^ cells in individual mice is shown. (C) No differences in T cell subpopulations were measured in blood of adult mice (12 weeks old). CD4^+^ and CD8^+^ T cells shown in A and C are also positive for CD3. Analyses were carried out in five independent experiments with MMP19^+/+^ (n = 4) and MMP19^−/−^ mice (n = 4). Numbers of B and T lymphocytes were analyzed by gating the lymphocyte region. Significances with *p<0.05; student's t-test.

Since differences in T cell populations between MMP19^−/−^ and wild-type mice were observed in the periphery and in lymph nodes, we were interested if the T cell population is also biased in the circulation. Blood of immature and mature unsensitized MMP19^−/−^ and wild-type mice were analyzed for the basic cell types. Analyzing T cell subpopulations we observed reduced numbers of CD8^+^ and CD4^+^ T cells in the blood of immature (3 weeks old) MMP19^−/−^ mice (Fig. 4A and B) whereas B lymphocytes (B220), monocytes (CD11b), and granulocytes (Ly-6) showed numbers comparable to MMP19^+/+^ mice. Analysis of 3 months old mice, i.e. adult mice, did not reveal any difference in T cell populations between MMP19^−/−^ and wild-type mice although the latter showed a tendency to increased amounts of CD4^+^ cells (Fig. 4C). Hence, the MMP19-deficiency influenced T cell proportions in the blood of immature mice.

### Thymocytes of immature MMP19^−/−^ mice exhibit decreased proliferation and reduced maturation

**Figure 5:**
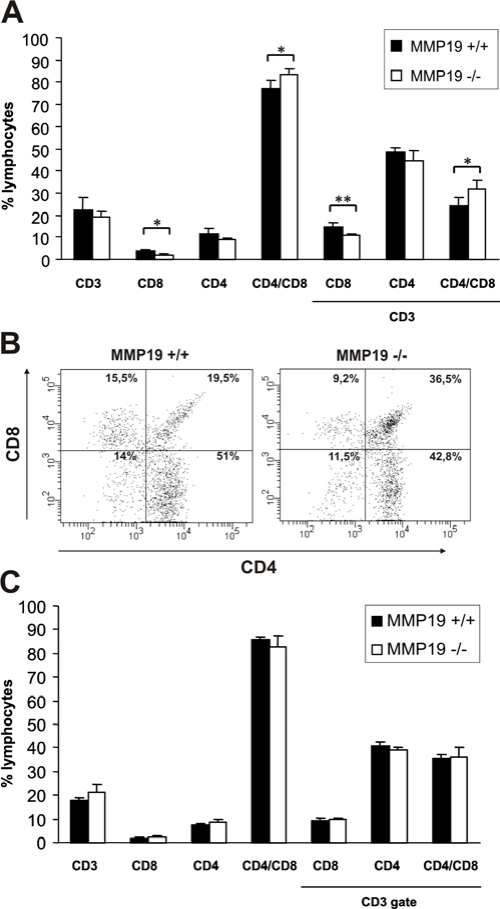
Development of single positive lymphocytes in the thymus of MMP19^−/−^ mice is impeded. Flow cytometry analysis of thymocytes from immature (3-weeks old, A and B) and adult (8–12 weeks, C) MMP19^+/+^ and MMP19^−/−^ mice. (A) Young MMP19^−/−^ mice have high numbers of CD4^+^/CD8^+^ T cells and lower numbers of CD8 single-positive thymocytes compared to MMP19^+/+^ mice. (B) A typical dot plot analysis of CD4^+^ and CD8^+^ T cell populations gated on CD3^+^ cells shows a decrease of single-positive and accumulation of double-positive T cells in thymi of MMP19^−/−^ mice. (C) Adult MMP19^+/+^ and MMP19^−/−^ mice show equal numbers of T cell subsets in the thymus. Analyses were done in four independent experiments with MMP19^+/+^ (n = 4) and MMP19^−/−^ mice (n = 4). Significant values with *p<0.05 and **p<0.01; student's t-test.

**Figure 6:**
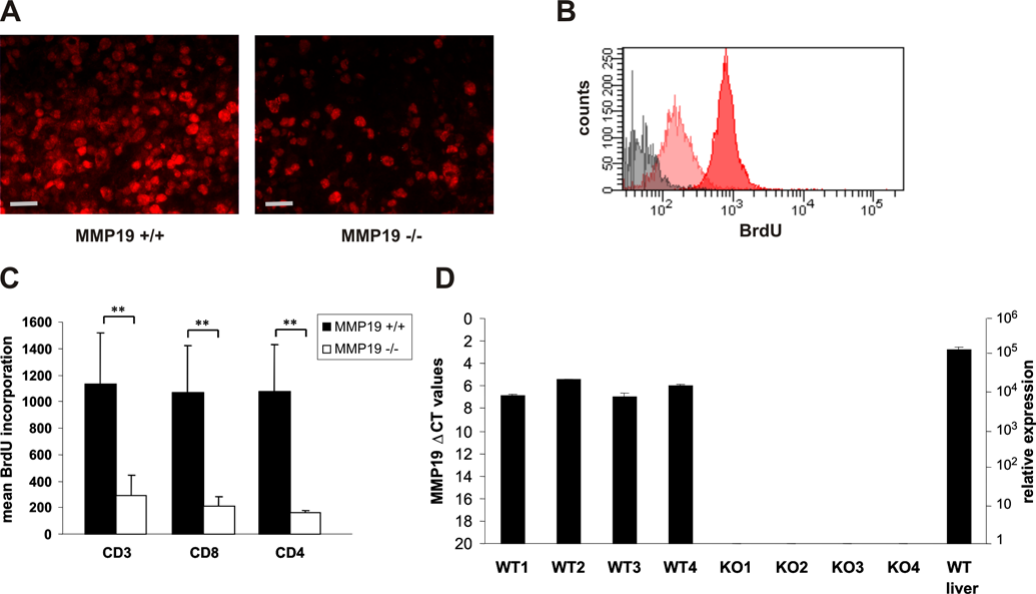
Thymocytes of MMP19^−/−^ mice exhibit strongly reduced proliferation. (A) Immature MMP19^−/−^ mice exhibit low numbers of Ki-67^+^ medullar cells in the thymus. Scale bars: 50 µm. (B and C). To quantify proliferating thymocytes mice were injected i.p. with BrdU. After 14 h, CD3^+^, CD4^+^, and CD8^+^ thymocytes were analyzed for BrdU incorporation using flow cytometry. Grey, isotype control; light red, MMP19^−/−^ (n = 4); red, MMP19^+/+^ (n = 4). (C) Quantification of BrdU positive thymocytes. CD3^+^, CD4^+^, and CD8^+^ thymocytes from MMP19^−/−^ mice exhibit markedly diminished proliferation. Black bars: MMP19^+/+^, white bars: MMP19^−/−^. (D) Quantitative RT-PCR analysis of MMP19 in the thymus shows expression in WT mice and confirmed the absence in MMP19^−/−^ mice. The expression of MMP19 in the thymus is lower compared to that in the liver. C_T_, cycle of threshold. Significances with **p<0,01 (student's t-test).

Searching for the origin of biased T cell population in blood, lymph nodes, and in peripheral tissues of MMP19^−/−^ mice we examined subset distribution of T cells in the thymus. Immature MMP19^−/−^ mice at the age of 3–4 weeks exhibit higher numbers of CD4^+^CD8^+^ DP cells and reduced levels of CD4^+^ and CD8^+^ SP cells compared to wild-type mice (Fig. 5A and B). In contrast, analysis of adult animals showed similar amounts of DP and SP cells in the thymus (Fig. 5C). This observation is in concordance with low amounts of SP T cells in blood of immature MMP19^−/−^ mice (Fig. 4). Furthermore, staining of thymocytes from 3-weeks old animals for the proliferation marker Ki-67 revealed low amounts of proliferating cells (Fig. 6A). These results were also confirmed *in vivo* by using BrdU incorporation. The incorporation of BrdU in thymocytes of MMP19^−/−^ mice was significantly reduced in comparison to MMP19^+/+^ mice (Fig. 6B and C). This altered proliferation in MMP19^−/−^ mice was not restricted to particular T cell subsets, since CD3^+^, CD8^+^, and CD4^+^ cells showed a similar degree of BrdU incorporation (Fig. 6C). To get a clue whether these changes could be directly mediated by the lack of MMP19 we studied the morphology of thymi from MMP19-deficient and wild-type mice and the expression of MMP19 using quantitative RT-PCR. Wild-type mice exhibited a low but defined expression of MMP19 in the thymus (Fig. 6D). Thus, MMP19 might be important for defining the thymic microenvironment or for migration of thymocytes among various niches. Analyzing the morphology of thymi from MMP19^+/+^ and -deficient mice we observed no differences in the cortical and medullar structure (not shown). Formation of the thymic vasculature was not altered, as the expression pattern of VE-cadherin, an endothelial marker, was comparable between wild-type and MMP19^−/−^ mice (not shown). Thus, MMP19 might influence the transition of DP thymocytes to SP mature T cells.

## Discussion

The present studies show that MMP19 plays a pivotal role during T cell-mediated cutaneous immune response and has an impact on the development of T lymphocytes. MMP19-deficient mice generated by disrupting its catalytic domain show no obvious morphological and developmental abnormalities. This is consistent with the work of Pendas et al. [59] who generated MMP19-deficient mice by replacing the promoter region and exons 1 and 2. Pendas and colleagues studied functions of MMP19 in metabolism, carcinogenesis, and angiogenesis and showed that MMP19-deficient mice are more susceptible for skin tumors and exhibit earlier tumor angiogenesis [59], [60]. We have studied MMP19 in the skin immunity and our results, together with the work on skin tumorigenesis show that MMP19 has indispensable functions in cutaneous homeostasis.

In the CHS model, the deficiency of MMP19 severely impaired the overall inflammatory response to the hapten suggesting a requirement of this MMP in cutaneous inflammation. A similar reduction of the inflammatory reaction in CHS was described also in stromelysin-1 (MMP3)-deficient mice while the lack of MMP9 resulted in the prolongation of the response. Interestingly, deficiency of MMP3 and MMP9 weakened also the immune reaction in the lung during acute injury although only MMP3 affected the influx of neutrophils [61]. Neutrophils are also controlled by MMP12 as MMP12-deficient mice exhibit a markedly reduced influx of neutrophils into the alveolar space during acute lung injury [62]. In general, the involvement of MMPs in immune reactions is only purely characterized, nevertheless studies with mice deficient in MMP9 and MMP7 showed that MMPs are essential for development of inflammation and its resolution (reviewed in Hu *et al*. [63]).

The lack of MMP19 not only considerably reduced the skin inflammation characterized by low influx of inflammatory cells as reported for MMP3-deficiency, but also reduced proliferation of keratinocytes and strikingly diminished activation of CD8^+^ T cells which are responsible for this type of reaction. This diminished activation was not due to an inability of DCs to migrate into LN and to present the hapten to T cells since the numbers and the activation state of DCs in MMP19^−/−^ and wild-type mice were comparable (not shown) in inguinal draining LNs.

With regard to the impaired inflammation it is possible that the amount of proinflammatory factors produced from the epidermis are not sufficient for the attraction and migration of T cells. This is also supported by markedly reduced concentrations of a number of cytokines including lymphotactin and I-TAC. Lymphotactin is a powerful attractant for T cells and is expressed by activated CD8^+^ T cells and by epidermal dendritic T cells affecting migration of CD8^+^ T cells. I-TAC, interferon-inducible T cell α chemoattractant (also known as CXCL11 or IP-9) that is also produced by keratinocytes binds to the chemokine receptors CXCR7 and CXCR3 and exhibits potent chemoattractant activity for interleukin-2-activated T cells [47], [48], [52], [64]. Since CD8^+^ T cells were less abundant at the inflammatory sites of MMP19^−/−^ mice than in control mice we suggest that the MMP19-dependent dysregulation of chemokines, such as lymphotactin and I-TAC, is responsible for the skewed distribution and circulation of T cells in the periphery and in LN. Thus, we suppose that proinflammatory factors produced by the inflamed tissue itself could contribute to T cell distribution and the skewed inflammatory reaction in MMP19^−/−^ mice.

The diminished production of inflammatory mediators correlated not only with reduced proliferation of keratinocytes in MMP19^−/−^ mice but also with markedly reduced processing of IGFBP-3 in keratinocytes. In our previous work we could show *in vitro* that MMP19-dependent proteolysis of IGFBP-3 leads to activation of IGF-signaling pathway and, thus, to increase of proliferation, production of proinflammatory factors, and cell migration [14]. Alterations of the immune response in MMP19^−/−^ mice not only point towards the role of MMP19 in controlling attraction and circulation of inflammatory cells but reveal also an option that T cell populations in MMP19^−/−^ mice might be generally biased. Indeed, the analysis of blood cells disclosed that CD4^+^ and CD8^+^ T cell populations are significantly diminished in immature mice. Reduced numbers of cells positive for CD3, CD4, and CD8 in the blood appear to be a direct consequence of the situation in the thymus. Thymi of immature MMP19^−/−^ mice exhibited increased numbers of CD4^+^CD8^+^ DP thymocytes while the number of CD8^+^ T cells was diminished. The numbers of CD4^+^ T cells were not significantly altered. Thus, thymocytes in immature MMP19 mice exhibit a delayed transition of DP cells into the SP state that is compensated with increased age. This distorted T cell development and biased T cell subpopulations in the blood are most likely not responsible for abrogation of the cutaneous inflammatory reaction since adult mice that were used for experiments did not exhibit these differences. Thus, MMP19 affects the local inflammation independently from developmental alterations of T cells.

MMP19 was previously reported to be abundant in human thymus [6] which is in line with its expression in mouse thymi. Although the underlying mechanism of MMP19 function in the thymus is currently not known, former studies with metalloproteinases (MP) inhibitors indicated possible functions of MP. They significantly reduced the numbers of CD4^+^CD8^+^ DP and mature SP T cells, inhibited thymocyte proliferation, and led to incomplete upregulation of CD25 (IL-2 receptor) [65]. MMP19^−/−^ mice showed both reduced proliferation of thymocytes and diminished numbers of CD25^+^CD8^+^ T cells during sensitization and effector phase of CHS. Although several MMPs are expressed in the thymus their specific role is unknown [55], [66], [67]. It was, however, suggested that MT1-MMP that is upregulated by thymocytes upon contact to laminin 5 contributes to thymocyte migration via releasing CD44 [55]. MMP19 might be also involved in adhesion, detachment, and migration of thymocytes since it processes several ECM proteins including type IV collagen, nidogen-1, and laminin 5 γ2 that were reported to be relevant for the migration and development of thymocytes [16], [17], [68]. However MMP19 is not the only protease that is able to process these substrates and, thus, cooperation of different MMPs and other proteases may lead to efficient processing of ECM proteins *in vivo*. The phenotypic changes that were found in MMP19-deficient mice could be rather a result of combined processing of multiple substrates than outcome of one major substrate. Moreover, we cannot exclude that beside IGFBP-3 processing, which is able to affect a number of cellular processes, MMP19 could also process and activate chemokines and proinflammatory cytokines as reported for other MMPs (reviewed in Van Lint P. and Libert C. [69])

Altogether, analysis of MMP19-deficient mice revealed that MMP19 plays an important role in the development of the cutaneous immune response, especially in CHS, and is involved in development of T lymphocytes, their distribution, and activation.

## Materials and Methods

### Targeted disruption of the *mmp19* gene

The targeting vector was designed to delete 1088 bp from the region spanning the end of exon 3 and the complete exon 4 containing the active site of MMP19. For construction of the 5′-arm of the targeting construct, a 2.8 kb fragment spanning a region including exon 2 and most of exon 3 was amplified from genomic DNA using primers m1U-2/*Eco*RI (GACTTCAGGCTGGAAGATATC) und m1D-5/*Eco*RI (AGATTTCTGGTTGAAGGG). The 3′-arm homology region of 2.8 kb ranging from exon 5 to exon 8 was created using primers m1U-25b/XhoI (TCATGGCTCCTGTCTATGCTG) and m1D-31/*NotI* (GAACACCTTTTGATTAACAGGC). The targeting construct pPNT/mmp19del was introduced into E14 embryonic stem cells [70]. For screening, DNA from G418/Gancyclovir-resistant colonies was digested with *Stu*I and *Eco*RV and assessed by Southern blotting using a nonoverlapping 3′-probe generated by PCR with primers derived from exon 8 (m1-U30, CGCTACCCTAAACCAATCAAG) and exon 9 (m1D-45, CAGGAATGTGGTATCCAGAAG). The targeting event was confirmed by Southern blot analysis using the strategy described above, i.e. probing with the 3′-probe led to identification of either a 7.3 kb band for the wild type or a 5 kb band for the targeted allele. Two clones, F6 and G5, were microinjected into blastocysts and chimeras were generated by standard techniques [71]. Upon germ line transmission, animals carrying the mutant *mmp19* allele were crossed with C57BL/6 mice and two mouse lines (F6 and G5) were established. Studies performed were done with mice backcrossed to C57BL/6 background at least to the F12 generation. Housing of mice and *in vivo* experiments were performed after approval by the Animal Care Committee of the University of Kiel in compliance with national and institutional guidelines.

### PCR analysis

Mice were genotyped using PCR. WT allele: forward primer in exon 4 (m1-U10, CCGCATCTTCAATGTGCCC) and a gene-specific reverse primer from exon 5 (mD1-21, GATGCGCAGGTTCACTCC) amplified a 800 bp fragment from the wild-type allele. Targeted allele: forward primer derived from exon 3 (m1-U3, AGATGGATGACGCCACAAG) and a reverse primer based on the PGK-Neo cassette (neofwd, CTTCTATCGCCTTCTTGACG) amplified a 600 bp fragment. Amplification of genomic DNA extracted from tail biopsies was performed using Taq DNA polymerase (Fermentas AB, Vilnius, Lithuania) and a program of 1 min at 95°C, followed by 30 cycles at 95°C (30 s), 60°C (30 s), and 72°C (1 min). RNA was isolated with TRIZOL reagent (Invitrogen, Carlsbad, CA). Quantitative PCR was performed using the ABI 7700 Prism Sequence Detection System and TaqMan primer probes specific for murine MMP19 and GAPDH (Applied Biosystems, Foster City, CA) as described [72]. Calculation of C_T_-values (cycle of threshold) were done as described [72].

### CHS model to FITC and irritant dermatitis to croton oil

CHS was carried out as described before [73], [74]. Briefly, on day 0 shaved mouse abdomens were painted with FITC (Sigma, Steinheim, Germany). After 5 days mice were challenged with FITC on both sides of one ear. Ear thickness was measured before challenge (0 h) and at 24, 48, and 72 h using a micrometer (Mitutoyo, Elk Grove Village, USA). DCs and T cells from draining lymph nodes were isolated 48 and 72 h after challenge and analyzed using flow cytometry. DCs were examined with antibodies against CD11c (BD Biosciences, San Jose, CA), CD80, CD86, and MHCII molecules (Caltag Laboratories, Burlingame). T cells were stained with antibodies against CD8, CD62L, CD69, CD44, CD122, and CD25 (BD Biosciences). DCs and T cells in inguinal lymph nodes were analyzed 24 h after sensitization. The model of irritant dermatitis was performed by application of croton oil (0.8% in acetone) onto mice ears. Ears were analyzed by immunohistochemistry 24 h after painting.

### Immunohistochemistry

Tissue sections were stained either with eosin and hematoxylin (Gill III, Thermo Shandon, Pittsburgh, PA) or with antibodies. Monoclonal anti-Ki-67 antibodies (clone Tec3, diluted 1∶50; Glostrup, Dako Cytomation) and polyclonal rabbit antibodies raised against a peptide derived from the hinge region of murine MMP19 was used. Bound antibodies were detected using the Vectastain ABC Elite kit and AEC as substrate (both Vector Laboratories, Burlingame, CA). For immunofluorescent detection tissues were embedded in tissue freezing medium (Leica Instruments, Nussloch, Germany) and monoclonal anti-CD8 (Chemicon, Temecula, CA) and anti-Ki-67 antibodies were used. Antibodies were detected with Cy3-conjugated goat anti-rat IgG antibodies (Dianova, Hamburg, Germany) and visualized by a BX-50 fluorescence microscope (Olympus, Tokyo, Japan). Images were processed with the Analysis Soft Imaging system (Soft Imaging System, Lakewood, CA).

### Isolation of primary keratinocytes and western blot analysis

Primary keratinocytes were isolated from newborn (day 1 to 2) mouse skin by trypsinization using a modified procedure as described previously [75] and grown in plastic vessels coated with type I collagen (10 µg/cm^2^) in keratinocyte medium (K-SFM) supplemented with EGF, BPE und 0,05 mM CaCl_2_ (Invitrogen, Carlsbad, CA, USA) at 37°C under 5% CO_2_. At confluency cell lysates were prepared as described [13], [14]. Protein was resolved on 10% SDS-polyacrylamide gels, transferred to PVDF membranes (Macherei-Nagel, Düren, Germany), and analyzed for MMP19 using polyclonal antibodies purified against a peptide derived from the hinge region of murine MMP19. IGFBP-3 processing was examined with polyclonal antibodies detecting full-length form and fragments of IGFBP-3 (Santa Cruz Biotechnologies, CA). Conditioned media of keratinocytes were 10-fold concentrated.

### Detection of cytokine expression

A mouse inflammation antibody array (Ray Biotech, Norcross, GA) was used according to the manufacturer's instructions. 24 h after CHS challenge inflamed ears were cut off and lyzed. A 500 µg aliquot of the tissue lysate was used for each array. Signal intensity was evaluated via AIDA Image Analyzer Software (Raytest, Straubenhardt, Germany).

### Isolation and FACS analysis of blood and thymus cells

Mice were anaesthetized and blood was obtained via intracardial punctation. After adding EDTA and washing cells with PBS erythrocytes were lyzed for 15 min at room temperature in lysis buffer (150 mM NH_4_Cl, 15 mM Na_2_CO_3_, 1 mM EDTA, pH 7.5). Thymi were excised immediately after the punctation. Cells were stained with anti-CD3, -CD4, and -CD8 antibodies (mouse T lymphocyte antibody cocktail, BD Biosciences). Blood cells were further stained against CD11b (Caltag), CD45R (B220), and Ly-6C/G (both BD Biosciences). Flow cytometry was carried out using a FACScanto (BD Biosciences).

### Thymocyte proliferation

To analyze proliferation by immunohistochemistry, sections of thymi were stained with anti-Ki-67 antibodies as described above. To quantify the proliferation mice were injected i.p. with 1 mg BrdU in 1ml PBS. After 14 h thymocytes were stained with antibodies against CD3, CD4, and CD8 (BD Biosciences). After fixation and permeabilization cells were incubated with FITC-conjugated anti-BrdU antibodies (Santa Cruz Biotechnologies, CA) and analyzed by flow cytometry.
